# Measuring the perceived wellbeing of hemodialysis patients: A Mind Genomics cartography

**DOI:** 10.1371/journal.pone.0302526

**Published:** 2024-05-13

**Authors:** Ermira Jahja, Petraq Papajorgji, Howard Moskowitz, Ioanna Margioukla, Fjona Nasto, Arjeta Dedej, Paola Pina, Mikel Shella, Manjola Collaku, Erjona Kaziu, Kristela Gjoni

**Affiliations:** 1 Faculty of Dental Medicine, Department of Basic Sciences, Western Balkans University, Tirana, Albania; 2 Proinfinit Consulting, Tirana, Albania; 3 World Institute of Competitive Excellence, New York, New York, United States of America; 4 Department of Psychology, American Hospital, Tirana, Albania; 5 Department of Nephrology, American Hospital, Tirana, Albania; 6 Diavita Dialysis Center, Elbasan, Albania; 7 Faculty of Technical Medical Sciences, Department of Medicine, Western Balkans University, Tirana, Albania; SRM Institute of Science and Technology, INDIA

## Abstract

Chronic Kidney Disease patients under hemodialysis have high morbidity rate, which tends to considerably affect their health-related quality of life. Multiple studies that have made use of different questionnaries report the poor life quality of this patient group. The research in hand implemented the Mind Genomics Approach as a method to asses the health-related quality of life of hemodialysis patients, while relying on conjoint measurements to group individuals with similar patterns of responses to a certain *mindset*. The study is conducted in 3 clinics with 219 patients. It uncovers three clusters or mindsets: Mindset 1- Feels guardedly optimistic but worried about money, Mindset 2—Feels strongly positive because the state guarantees and the family supports, Mindset 3—Feels positive only about money. Based on the analysis of the collected data, the findings of this study suggest that the quality of life in hemodialysis patients is highly correlated to their financial status. The current study is one of the few first attempts to apply Mind Genomics in medical settings and the first, to our knowledge, in hemodialysis centers. This technology might enable healthcare proffesionals to provide personalized psychological treatment and additional social support to patients, which in turn could improve their clinical outcomes. The study is an example of using technology as a service.

## Introduction

Dialysis is a method which partly substitutes the role of kidneys, by the maintenance of water balance and the excretion of waste products arising from protein metabolism. Chronic Kidney Disease (CKD) is the condition referring to the gradual loss of kidney function, defined by Glomerular Filtration Rate (GFR) <60 mL/min/1.73 m^2^ for at least 3 months, what is commonly caused by diabetes, high blood pressure and glomerular diseases. Due to a weakened immunity system and the overall cardiovascular risk, CKD patients are among the most vulnerable chronic patients [1]. Therefore, as the condition advances, the patient must initiate the dialysis therapy. The average life expectancy of these patients is 5–10 years, although dialysis treatment has been reported to last more than 30 years [2,3]. CKD cases are represented in a series of stages, starting from 1—Not Severe—up to 5—End-Stage Renal Disease: ESRD -, of which stage 5 refers to patients bound to maintenance dialysis [4]. The main treatment modalities for CKD are hemodialysis and peritoneal dialysis, each of which can be provided in-center or at home. Advances in dialysis have increased the expectations for this category of patients, from simple survival to achieving a certain level of wellbeing [5]. The age of patients on dialysis ranges from pediatric cases to elders, with a global trend of increase in the percent of end-stage patients older than 65 [6].

The current global prevalence of hemodialysis patients is estimated to be 5.3 to 9.7 million, the majority of whom live in upper-middle income countries [7]. The number of patients receiving dialysis is increasing rapidly in low and middle-income countries, as a result of the increase in the prevalence of hypertension, diabetes mellitus, and exposure to environmental pollutants [8]. Yet, access to dialysis is still limited, resulting in millions of deaths from kidney failure each year.

The mortality rate and comorbidities among patients receiving dialysis are high, and this usually has considerable effects on their Health-Related Quality of Life (HRQoL) [7,9–11]. Another issue is the perceived quality of life, which refers to people’s perception towards their life, their life experience, and their subjective feeling of well-being [12]. This paper focuses precisely on the perceived health related-quality of life among these patients. HRQoL, is defined as a multidimensional assessment of how diseases and treatments affect the perception of patients on their wellbeing during or after treatment, based on physical, psychological and social aspects of life. Applied extensively on cancer patients, HRQoL elements have shown more accurate prognostic efficacy than socio-demographic and clinical characteristics alone [13].

Among the most common approaches applied to assess the HRQoL in dialysis patients are: Kidney Disease Quality of Life Short Form-36 (KDQoL-SF 36) [14,15], EQ-5D [16] and Self Evaluated Individualized Quality of Life (SEIQoL) [17]. Apart from KDQoL-SF 36, which is applied particularly for kidney-disease research, the other two approaches are used in other clinical studies as well as by the industry.

The most common way to collect and assess people’s perceived condition, are questionnaires or surveys, which typically are made up of close-ended questions and require the selection of a single choice among pre-defined responses. However, recently, the focus has moved beyond simple questionnaires, which tend to be regarded as biased, because respondents often attempt to ’guess’ the correct or appropriate answer. More innovative surveying approaches that ask respondents to evaluate combinations or mixtures of ideas, have become more frequent. These new approaches to surveys analyze responses to create patterns that reveal the respondent’s opinion and reduce or totally eliminate the potential biases that can play out when the test is based on the selection of single answers. The respondent cannot determine the ’right answer’, thus, he is expected to be answering honestly and to be using a single ’mental scale’ to evaluate the different test combinations rather than unconsciously adjusting the scale to fit the specific question. The new method is generically known as "conjoint measurement" [18], differing dramatically from the bias-prone ’one-at-a-time’ methods.

In compliance with the criteria of less biased and more revealing surveying methods, Mind Genomics is an approach based on conjoint measurements [19]. Mind Genomics study aim is to uncover mindsets’ patterns and to, thus, demonstrate what pushes people to make decisions. These patterns emerge from analyzing the combinations of ideas created using experimental design techniques [20]. Respondents evaluate the combinations, after which Ordinary Least-Squares (OLS) regression is used to deconstruct the responses to the contribution of each of the involved elements. The final analysis in Mind Genomics uses the statistical method of clustering [21] to group individuals with similar patterns of responses.

The actual mechanics of Mind Genomics is straightforward. The researcher identifies an area of interest—the overall topic and defines a set of four questions or *silos*, which will serve as the pillars of the study [22]. Afterwards, four answers that would cover the entire spectrum—positive, neutral, and negative—are determined for each question. Thus, the model used is called the 4X4 model. An underlying experimental design combines these elements into small ‘vignettes’ comprising 2 to 4 answers [23,24]. The response to these vignettes, deconstructed by regression, shows the contribution of the specific elements [25]. At a more profound holistic level, Mind Genomics reveals patterns of responses, suggesting ‘mindsets’, that is, different ways of organizing the information.

From the beginning of the century, Mind Genomics has been applied in different areas, such as consumer behavior and choice analysis [26,27], in the food industry [28–30], and in psychology [31]. In addition, it has also been used in law [26] and in the analysis of social and political issues [32,33]. Mind Genomics is an example of the use of technology as a service. A web-based application (https://www.bimileap.com/) guides the use of this technology.

As Mind Genomics continues to evolve, it is given increased attention from healthcare professionals and reserachers [34,35]. The reasons for this interest are three distinct factors. The first factor is granularity. Mind Genomics presents respondents with combinations of messages that are easy to understand, concrete (authentic), and specific. The respondent is presented with vignettes written in everyday language. Respondents understand the granularity—the specificity—and find it easy to evaluate the vignettes. The second factor is the ease and low cost/low risk to iterate, with these studies taking the respondents few minutes to complete and the analyses being ready in minutes after the last respondent has finished answering. The third and most important factor that contributes to the increased popularity of Mind Genomics studies is the Personal Viewpoint Identifier (PVI). PVI is a set of six questions that use elements which are the same as in the study, to assign non-participants to an existing mindset. A significant advantage of the PVI is that it allows for continuous addition of new participants to the already defined mindsets [30], thus, increasing the sample size at a very low cost. This mode of communication is a promising future approach, offering better predictive models that would, in turn, provide better healthcare services and particularly contribute to personalized healthcare.

A distinctive advantage of the PVI is that the assignment of any individual to the existing mindsets is not made based upon who the person is, but rather on how the person thinks about a certain issue. This feature of the Mind Genomics PVI is critical because the alignment of any individual in a mindset is predicted from relevant measures of mindsets for a particular issue. As a result, two people who are exact ‘look alike’ in terms of their behavior and geo-demographics, may actually be members of different mindsets, and require different interaction patterns.

Taking these into consideration, this study analyzes the perceived wellbeing of patients under maintenance dialysis. It researches how dialysis patients evaluate their state of wellbeing. In other words, it attempts to elucidate the “mindsets” of this patient category. The mindset reveals itself at the granular level. The participants’ responses to the questions define their well-being or ‘mindset’.

There is a vivid discussion in the field literature in relation to whether hypothesis-based or hypothesis-free methods are more appropriate for scientific studies. Each of these approaches is extensively supported by different researchers [36–39]. The fast and cheap acquisition of massive amounts of data has brought hypothesis-based studies into question, since it has become apparent that every bit of the collected data might hold vital information about the topic under scrutiny [39,40], and focusing on predetermined notions could prevent researchers from looking into other patterns the collected data may contain [40]. For this reason, the research herein maintains a hypothesis-free approach, so that every possible implications of each of the included elements is considered and not missed.

## Methods

This study was conducted in three dialysis facility centers situated in Albania: American Hospital of Tirana 1, American Hospital of Tirana 2 and Diavita Elbasan. The questionnaires were administred from January 27, 2021 to February 4, 2022. The consent of all the participants in the study was obtained in an electronic format. The study was carried out in accordance with the Albanian Code of Medical Ethics and Deontology, and Diaverum Code of conduct.

The study focused on the responses of dialysis patients to messages—elements—of a general nature, yet all related to the four aspects of life quality. The four aspects that were included in the questionnaires as questions or silos and that reflect life quality are: family support, health system assistance, income and future perspective. As it will be shown below, the questionnary silos were selected to be elucidating in relation to the analyzed issue, yet sufficiently general to provide a basis of comparison across the groups, which were created on the basis of the individulas’ declared age, gender, and number of years under dialysis.

Mind Genomics works in a structured fashion. The first step involves the selection of the issue or topic that will be studied. The topic of this study was defined as: ‘The Points of View of the Dialysis Patients’. The second step consists of the compilation of four questions—or silos—which ‘tell a story’, or at least can be the basis of a story. The four aspects above (family support, health system assistance, income and future perspective) underline the four questions needed for the Mind Genomics system. The third step consists of providing four specific answers to each of the questions. Considering that this is an early-stage study, the involved group of researchers decided to provide four simple, graded statements or answers to each question, as shown in Table 1.

**Table 1:** The four silos and the corresponding elements.

**Silo A. ECONOMICS: How do you describe your financial condition?**	
A1	I’m in a very good financial condition
A2	I have above average finances
A3	I have some economic problems
A4	I struggle to afford life economically
**Silo B: MEDICAL SUPPORT: Are you happy with the medical support? (Does the medical system support you?)**	
B1	The free medical system covers all my medical needs
B2	The free medical system covers most of my dialysis treatment and most of the other medical needs
B3	The free medical systemcovers most of my dialysis treatment but only few of other medical needs
B4	I am provided no support by the state health system
Silo C: **FAMILY: Do you have family support? (How much does your family support you?)**	
C1	My family supports me significantly
C2	My family supports me most of the time
C3	My family supports me only partially
C4	I don’t have any support from my family
Silo D: **FUTURE PERSPECTIVE: How do you feel about your future?**	
D1	I feel very encouraged about my future
D2	I feel my future might be somehow positive
D3	I feel my future might be somehow negative
D4	I feel hopeless for the future

The experiment begins with the use of an experimental design to come up with the combinations, also referred to as “vignettes” [41]. The experimental design specifies the precise combinations of 24 vignettes, set up so that a vignette comprises at least two answers and, at most, four elements. Additionally, the vignette cannot comprise more than one element from a silo, in other words, an answer for a question. However, the experimental design demonstrated that a considerable number of vignettes was incomplete, lacking elements from one or two silos. The experimental design ensures that the 16 elements appear exactly five times within the set of 24 vignettes: that is, 20 out of 24 vignettes comprise the very same element of one silo and that the other four vignettes do not include any elements from that silo. This design also ensures that the 16 elements are statistically independent from each other, allowing the use of regression analysis at the level of a single respondent. Thus, a participant in the survey provides 24 independent evaluations.

A vital feature of the Mind Genomics system is the permutation of the basic experimental design. The mathematical structure of the design is maintained, while the composition of the 24 vignettes changes. The result is that each respondent evaluates a different combination of vignettes. Mind Genomics approach can be considered an exploratory system, in the same fashion with the Magnetic Resonance Imaging (MRI), which takes pictures of the same item from different angles, to have these pictures combined afterward with the help of a computer algorithm, and generates a detailed three-dimensional view of the underlying item. In compliance with the MRI functioning mode, the results obtained by each respondent can be considered as a ‘picture’ and their combination will generate a detailed and relatively more reliable view of the situation. In other words, the benefit of such an approach is a deeper understanding of the mindset of the group of individuals participating in the study [18].

The Mind Genomics method works at the level of ’granular information’: that is, information that creates a picture with words for the respondent. The interaction is defined as the researcher’s use of text phrases, wich could be defined as the raw material and which, in turn, are formed by the answers from the questions—elements from silo -, and the responses to the combinations of these phrases presented in the vignettes. The research strategy identifies the different mindsets, if there are more than one. These mindsets emerge from how people with the condition respond the questions related to their health and wellbeing in their daily lives.

As noted above, each respondent in the study evaluated a different set of 24 vignettes, each vignette in the group was specified by the underlying experimental design. Rather than having one basic experimental design, the Mind Genomics system assigned each respondent to a different permutation, a different set of 24 combinations. In this way, the Mind Genomics approach does not have to somehow ‘guess’ the appropriate elements on which the study will focus. On the contrary, the data themselves, that is, the coefficients from the analysis of the individual level respondent, will point out the significant elements and show the underlying mindsets emerging from them. The rest of the data which consist of insignificant elements, can be discarded or at least ignored.

The total number of respondents is 219 dialysis patients (Supplementary material) who are treated at 3 different hospitals in Albania. If necessary, the patients who participated were assisted by the medical staff and a psychologist who was the one to read the combinations and reminded the patients about evaluating the vignette as a whole. The respondents withdrew from the study when judged necessary by the medical staff members or by patients themselves.

A total of 5,256 (219 x 24) observations were gathered for this study.

The respondents were introduced to the study by the paragraph reproduced below:

*This study aims at evaluating the wellbeing of a dialysis patient*. *Please consider the entire context presented in the vignette as one and evaluate the vignette*. *Sometimes it may look that there are repetitions but there are not*. *Vignettes contain information that may be similar but not the same*. *Please consider the vignette as one contextual entirety*!

The analysis proceeded in six steps.

Step 1 focused on the creation of the underlying database, with each respondent generating 24 rows of data, one row for each of the evaluated vignettes, and 16 columns to code the presence/absence of the elements.‘1’ denotes the element was present in the vignette, and ‘0’ shows that the element was absent. The 17^th^ column was the 9-point rating. The other columns coded the respondent identification number, age, gender, and information about their dialysis treatment. All coding was done so that the respondents’ privacy was preserved.
Step 2 consisted in the transformation of the rating from a 9-point Likert scale [42], anchored at both extremes, to a binary scale. The ratings from 1 to 6 were transformed to “0” to denote “Unlikelihood”. Ratings from 7 to 9 were turned into 100 to denote “Likelihood”. The transformation was done in line with Mind Genomics studies [43], which are presented to managers who need to understand the meaning of the scale values. Although most managers understand statistics well, they are not capable to make sense of the average numbers of scale values in terms of practical steps. For this reason, the switching of the data to binary no/yes or 0/100 makes their interpretation much easier.
Step 3 made use of only the two positive statements found in each silo—answer A1 A2, B1 B2, C1 C2, and D1, D2—as the independent variables in the OLS regression analysis [44]. The rationale for this selection was based on the idea that the remaining elements provided little information and could be confusing when the regression was interpreted. The selection of the first two positive statements was statistically appropriate at the level of each respondent, because each respondent evaluated all the 24 set up vignettes as a valid experimental design. The dependent variable was defined at the newly transformed binary variable.
Step 4 consisted in computing the OLS regression at the level of each of the 219 respondents. The regression is put through the origin, so the regression equation is expressed as: Binary Response = k_1_A1 + k_2_A2 + K_3_B1 + k_4_B2 + k_5_C1 + k_6_C2 + k_7_D1 + k_8_D2. The 10 sets of eight ‘zero coefficients’, which are those showing no variation in the newly created binary variables, were eliminated from further analysis.
Step 5 consisted in the creation of clusters—mindsets—of respondents, showing similar patterns of the eight coefficients computed in Step 4. The clustering [21] was done by k-means clustering, with the measure of distance between pairs of respondents expressed as “1-Pearson R”, with Pearson R computed using the eight coefficients for each respondent as the input. The clustering emerged with three definable clusters [45].
Step 6 consisted in the estimation of the eight coefficients for each definable subgroup; the subgroups were indentifiably named as “Total”, “Gender”, “Age”, “Self-Defined Attitude toward Dialysis”, while the other subgroups were the three emergent mindsets (Supplementary material).

## Results

As previously highlighted, the experiment uses a 9-point Likert scale to evaluate the respondent’s answers (Supplementary material). A 9-point Likert scale is transformed into a binary evaluation system to facilitate the data treatment. Thus, evaluations from 1 to 6 are considered negative and are represented by 0; evaluations from 7 to 9 are considered positive and are represented by 100. Then, the statistical models are executed with these new evaluation data. Table 2 shows the results obtained when the evaluations, including the ones evaluated with 7, 8, and 9, are considered positive.

**Table 2:** Performance of the elements and mindset generation.

	**RATE987 (Top 3 –Applies to me)**	**Total**	**Male**	**Female**	**Age 30–60**	**Age 61+**
**S1**		209	121	88	93	113
A1	I am in a very good financial condition	6	6	7	7	7
A2	My financial situation is above average	8	8	8	7	8
B1	The state health system covers all my medical expenses	7	6	9	9	6
B2	The state health system covers most of my dialysis treatment and most of other medical expenses	7	6	8	8	5
C1	My family strongly supports me	8	8	8	7	9
C2	My family supports me most of the time	9	12	6	12	7
D1	I feel very encouraged about my future	9	7	10	6	11
D2	I feel my future might be somehow positive	6	5	6	7	5
			**Number of years in dialysis**			
	**RATE987**	**Total**	**0–1 Years**	**1–5 Years**	**5–10 Years**	**10+ Years**
**S2**		209	38	87	61	23
A1	I am in a very good financial condition	6	9	6	7	2
A2	My financial situation is above average	8	7	9	7	6
B1	The state health system covers all my medical expenses	7	10	8	6	3
B2	The state health system covers most of my dialysis treatment and most of other medical expenses	7	8	8	4	3
C1	(C1: My family strongly supports me)	8	5	10	6	8
C2	My family supports me most of the time	9	8	9	9	12
D1	I feel very encouraged about my future	9	5	10	11	1
D2	I feel my future might be somehow positive	6	2	8	5	4
	**How the different elements drive the binary rating computed from ratings 9,8,7**	**Total**	**MS1–Feels guardedly positive but worried about money**	**MS2–Feels strongly positive because the state guarantees and the family supports**	**MS3 –Feels positive only about money**	
**S3**		209	71	79	59	
D1	I feel very encouraged about my future	9	25	-5	7	
C2	My family supports me most of the time	9	13	16	-5	
D2	I feel my future might be somehow positive	6	12	-4	12	
C1	My family strongly supports me	8	9	14	-1	
B2	The state health system covers most of my dialysis treatment and most of other medical expenses	7	5	13	2	
B1	The state health system covers all my medical expenses	7	4	10	8	
A2	My financial situation is above average	8	-5	8	22	
A1	I am in a very good financial condition	6	0	7	14	

The strongest and weakest elements for hemodialysis patients, based on the gender and two age groups (upper section S1), number of years in dialysis (middle section S2), and the generated mindsets (bottom section S3).

Considering the respondent’s gender, the strong elements are 12 for male and 10 for female respondents. Thus, men seem to have higher family support (C2: My family supports me most of the time), and females feel more secure about the future (D1: I feel very encouraged about my future). Similarly, considering the respondent’s age, the strong element is 12 for the group aged 30 to 60 (C2: My family supports me most of the time) and 11 for the group over 61 (D1: I feel very encouraged about my future.)

Considering the “number of years in dialysis” criterion, Table 2 shows that the strong value for people attending the dialysis process for the first year is 10 (D1: The state health system covers all my medical expenses). For the group of people being treated with dialysis from 1 to 5 years, the strong value is 10; it corresponds to element C1 (My family supports me significantly). The study demonstrated that for the group of people being treated with dialysis between 5 and 10 years, the strong value is 11, corresponding to element D1 (I feel very encouraged about my future). The strong value of the group treated with dialysis for over 10 years, is 12, corresponding to element C2 (My family supports me most of the time).

Mind Genomics creates mindsets, groups, or clusters of people who think or react to the stimuli similarly [33]. It uses clustering technologies [21] to group people of similar judgments. In order to achieve this, besides the statistical definition, Mind Genomics also imposes the following criteria [29]:

1. Interpretability–The clusters must make intuitive sense and tell a reasonably clear story. The meaning of the clusters comes from the commonality of the elements which show the highest coefficient.
2. Parsimony–Fewer clusters are always better than more clusters. It may be better to work with fewer clusters, even at the cost of losing some interpretability.

Following the statistical approach of k-means [21], and the additional selection criteria for the clustering, this study came up with three clusters—or mindsets—as presented in Table 2. Mindset 1 is designated as MS1 –Feels guardedly optimistic but worried about money. It is created by grouping elements D1 (I feel very encouraged about my future), C2 (My family supports me most of the time), and D2 (I feel my future might be somehow positive).

Mindset 2 is designated as MS2 –Feels strongly positive because the state guarantees and the family supports. It is created by grouping elements C2 (My family supports me most of the time), C1 (My family supports me significantly), B2 (The state health system covers most of my dialysis treatment and most of other medical expenses), and B1 (The state health system covers all my medical expenses).

Mindset 3 is designated as MS3 –Feels positive only about money. It is created by grouping elements D2 (I feel my future might be somehow positive), A2 (My financial situation is above average), and A1 (I am in a very good financial condition).

## Discussion

Mind Genomics is a powerful instrument to conduct holistic analyses in different areas of human activity. Mind Genomics enables researchers to use up to ten variables,—like age, gender, and other variables of researchers’ choice—to discover patterns in the collected data that may not be visible. At the end of the study, Mind Genomics discovers two or three mindsets that group respondents which think alike in one mindset. Thus, the mindsets provide the main issues that must be addressed.

In comparison to what Mind Genomics offers, existing approaches which measure the HRQoL by classical questionnaires are time-consuming and mainly applied for research purposes. However, they fail to provide the holistic perception of patients in relation to their wellbeing and, thus, cannot adequately offer personalized support.

Mind Genomics has been successfully used in several studies, such as human relations [46], tourism [23,47] banking [23], legal settings [24], social and political sciences [32], new teaching paradigms [48] and corruption in education [49], yet the attempts to benefit from it in the field of medicine and healthcare are few.

Mind Genomics is offered as a service online that allows researchers of different fields, to use modern technology at a very low intellectual effort and financial cost. Furthermore, its PVI feature creates an open system, enabling researchers to continuously add new participants to the study without having to go through the entire system again and again. Thus, it is possible to increase the sample size with no additional cost. It should also be noted that PVI applied in hospitals enables healthcare proffesionals to provide personalized psychological treatment and additional social support, which, in turn, could improve the clinical treatment offered to patients.

A recent (2020) study suggests that the predictive analytics using the Mind Genomics PVI might “promote health by predicting clinical outcomes for individual patients” [50]. The data used for this PVI came from a study in which 200 customers of the same insurance program were presented with different vignettes, systematically varied by design. The respondents rated each vignette on two scales. The first scale was interest in the insurance program while the second scale was a selection on how much the respondent would pay for the particular vignette describing the insurance program. The combination of the two scales revealed the respondent’s opinion in terms of what the respondent wants, but also the dollar value of each feature in the insurance. These and other similar data are very promising when it comes to the incorporation of the patient’s’ “mind” in the establishment of future personalized health plans. In addition, these initiatives could assist the healthcare system to better invest in preventive medicine.

Another study on healthy individuals using Mind Genomics focused on measuring their concern in relation to the prospect of cancer [35]. It pointed out to the existence of two distinct mindsets: one group perceived cancer as a chronic disease and the other feared the possibility of not recovering and dying. Mind Genomics was also applied to assess the emotional wellbeing of hospitalized teens. It revealed either a positive, or a very negative viewpoint of this age group regarding the hospital visit [51].

The study at hand focused on the perceived wellbeing of patients under maintenance dialysis, as this group of patients is amongst the most vulnerable, with a mortality rate 25 times higher than that of the normal population [52]. It is one of the first efforts to implement Mind Genomics in medical centers. Thus, the language of the answers was kept fairly ‘sterile’ and straightforward, as a way of finding out and researching the mindsets that can be gleaned by responses to simplistic elements. This way of cummunication does also reduce the influence that demographic, cultural and educational background of participants might have on responses.

As with other surveying technologies, Mind Genomics can be impacted by the phenomenon known in the terminology of political sciences as "courtesy bias." Respondents may need to be more well-focused during the experiment, or they may rush to finish the survey. Mind Genomics collects the data through vignette evaluation, a new approach to surveying technology. Thus, the respondent is prompted to evaluate a new context each time and must spend some time reading and understanding the vignette. It is unlikely, we believe, that the respondent will rush through the answers as in the case of yes/no type of surveys. The audience of this study is dialysis patients, and we believe them to be responsible and prudent while answering the interviews; the study results directly involve them.

This study aims at evaluating the well-being of the patients under dialysis and to understand what dialysis patients think about the state health system, the problems they face and, based on the findings, make suggestions for the improvement of the system.

Several factors that significantly influence the wellbeing of hemodialysis patients were examined. Multiple studies have demonstrated that in comparison to the general population, dialysis patients have a poorer life quality, which is mainly the result of the psychosocial and socioeconomic factors [53] and which manifests itself with depressive thinking and anxiety [54,55]. Also in comparison to non-depressed individuals, time perspective, as an indicator of emotional health, is reported to be significantly altered in depressed individuals who also tend to have a pessimistic attitude for the future [56].

These valuable insights were meticulously considered while the questionnaire was prepared. After it was administered, the obtained results demonstrated that the respondents can be divided into three mindsets. The first mindset, MS1 –“Feels guardedly optimistic but worried about money”, could be related with the high unemployment rate among ESRD patients. Hemodialysis regimen significantly increases the risk of unemployment due to the dialysis schedule. Furthermore, comorbidities in ESRD patients also contribute to unemployment due to work disability and frequent hospitalizations [57,58]. The other mindset, MS2 –“Feels strongly positive because the state guarantees and the family supports”, shows a general note of optimism in this group of patients. Such perceptions could be explained by several factors in the context of the Albanian culture and health system advancements. Strong family bonds and the culture of caring for the sick in the community probably have a high contribution to the perceived wellbeing of hemodialysis patients in MS2. In Albania, during the last decade, a public-private partnership provides free healthcare service to hemodialysis patients [59]. Such reimbursement services help to sustain treatment, which, in turn, alleviates both physical and psychological stress among hemodialysis patients and somehow compensates for the loss of productivity [60]. The importance of finances for the respondents is revealed in MS3—“Feels positive only about money”. This patient group seems to care and be encouraged only by money.

Due to the economic burden hemodialysis treatment implies, patients seem overall satisfied by the dialysis service and reimbursement policies. Although the three mindsets are different groups, they have a note of optimism altogether and a positive sense for the present and the future. Financial considerations are evident in all the three mindsets, which could possibly imply a general concern about financial self-sufficiency. Implementation of the present Mind Genomics platform in hemodialysis patients would require patients assigned to MS1 to be offered adequate employment opportunities.

## Conclusion

In conclusion, the three mindsets revealed in the study at hand suggest that Mind Genomics could be implemented in healthcare settings, such as hemodialysis centers and beyond. Mind Genomics allows for the defining of up to ten variables, enabling researchers to get a holistic understanding of the problem under study. Thus, researchers in this study clearly understand how dialysis patients react to this condition based on age, gender, and years of treatment.

The current study and similar Mind Genomics research would help communicate effectively with patients and improve their health outcomes. Mindsets can assist the personalized psychological treatment and social support of patients, thus increasing the potential for patients to receive adequate treatment, especially the most vulnerable ones detected by Mind Genomics system.

The herein study should be considered as a preliminary step towards a better understanding of the dialysis patient mindset. Further studies are warranted to define whether the patients’ socioeconomic and cultural backgrounds affect the emerging mindsets. Additional investigations would also reveal ways to better communicate with ESRD patients and alleviate the burden of hemodialysis.

## Supporting information

S1 File Raw data, original ratings of elements and mindset summaries presented into multiple worksheets. (XLSX)

S1 Table Mindset summary (top down). (PDF)

S2 Table Mindset summary (bottom up). (PDF)
