# Supplementary material for: Measuring the perceived wellbeing of hemodialysis patients: A Mind Genomics cartography
Source: PLoS One. 2024 May 13;19(5):e0302526. doi: 10.1371/journal.pone.0302526 (PMC11090323; doi:10.1371/journal.pone.0302526)
Supplement: S1 Table — (PDF) [file pone.0302526.s002.pdf]

S1 Table: Mindset Summary (Top Down)

Group (Binary Ratings)	Total	Segment 1 of 2	Segment 2 of 2	Segment 1 of 3	Segment 2 of 3	Segment 3 of 3
**Base Size**	219	109	110	84	76	59
**Additive Constant**	24	23	27	21	23	30
**Question A: Economics: How do you describe your financial condition**						
I am in a very good financial condition					3	
My financial situation is above average			5		6	
I have some economic problems			5		6	
Economically I struggle to afford life			1		4	
**Question B: Medical Support: Are you happy with the medical support**						
The state health system covers all my medical expenses				2		
The state health system covers most of my dialysis treatment and most of other medical expenses					2	
The state health system covers most of my dialysis cost but only few other medical expenses				4	4	
No support is provided by the state health system				1		
**Question C: Family: Do you have family support**						
My family strongly supports me						3
My family supports me most of the time			5		2	6
My family supports me only partially			1		1	
I do not have any support from my family						1

**Question D: Future Perspective: How do you feel about the future**						
I feel very encouraged about my future		8		6		10
I feel my future might be somehow positive		6		5		2
I feel my future might be somehow negative		7		8		1
I feel the future is hopeless		8		11		
**Group (Binary Ratings)**	**Total**	**Segment 1 of 2**	**Segment 2 of 2**			
**Base Size**	219	109	110			
**Additive Constant**	24	23	27			
**Strong for Mind-Set 1**						
I feel very encouraged about my future		8				
I feel the future is hopeless		8				
**Strong for Mind-Set 2**						
**Not strong for either mind-set**						
I am in a very good financial condition						
My financial situation is above average			5			
I have some economic problems			5			
Economically I struggle to afford life			1			
The state health system covers all my medical expenses						

The state health system covers most of my dialysis treatment and most of other medical expenses						
The state health system covers most of my dialysis cost but only few other medical expenses						
No support is provided by the state health system						
My family strongly supports me						
My family supports me most of the time			5			
My family supports me only partially			1			
I do not have any support from my family						
I feel my future might be somehow positive		6				
I feel my future might be somehow negative		7				
**Group (Binary Ratings)**	**Total**			**Segment 1 of 3**	**Segment 2 of 3**	**Segment 3 of 3**
**Base Size**	219			84	76	59
**Additive Constant**	24			21	23	30
**Strong for Mind-Set 1**						
I feel the future is hopeless				11		
I feel my future might be somehow negative				8		1
**Strong for Mind-Set 2**						

**Strong for Mind-Set 3**						
I feel very encouraged about my future				6		10
**Not strong for either mind-set**						
I am in a very good financial condition					3	
My financial situation is above average					6	
I have some economic problems					6	
Economically I struggle to afford life					4	
The state health system covers all my medical expenses				2		
The state health system covers most of my dialysis treatment and most of other medical expenses					2	
The state health system covers most of my dialysis cost but only few other medical expenses				4	4	
No support is provided by the state health system				1		
My family strongly supports me						3
My family supports me most of the time					2	6
My family supports me only partially					1	
I do not have any support from my family						1
I feel my future might be somehow positive				5		2
