# Supplementary material for: Measuring the perceived wellbeing of hemodialysis patients: A Mind Genomics cartography
Source: PLoS One. 2024 May 13;19(5):e0302526. doi: 10.1371/journal.pone.0302526 (PMC11090323; doi:10.1371/journal.pone.0302526)
Supplement: S2 Table — (PDF) [file pone.0302526.s003.pdf]

S2 Table: Mindset Summary (Bottom Up)

**Group (Binary Ratings)**	**Total**	**Segment 1 of 2**	**Segment 2 of 2**	**Segment 1 of 3**	**Segment 2 of 3**	**Segment 3 of 3**
**Base Size**	219	109	110	84	76	59
**Additive Constant**	44	47	41	48	46	37
**Question A: Economics: How do you describe your financial condition?**						
I am in a very good financial condition	5	11		12	1	
My financial situation is above average	3	9		9		
I have some economic problems	3	9		9		
Economically I struggle to afford life	5	10	1	11	2	1
**Question B: Medical Support: Are you happy with the medical support?**						
The state health system covers all my medical expenses	1	1	1			11
The state health system covers most of my dialysis treatment and most of other medical expenses	3	2	4			16
The state health system covers most of my dialysis cost but only few other medical expenses		1				10
No support is provided by the state health system	3	2	4	1		10

**Question C: Family: Do you have family support?**						
My family strongly supports me	1		4		4	
My family supports me most of the time	1	3				1
My family supports me only partially	2	2			1	4
I do not have any support from my family	1		1		3	1
**Question D: Future Perspective: How do you feel about the future?**						
I feel very encouraged about my future	1		7		11	
I feel my future might be somehow positive	1		7		10	
I feel my future might be somehow negative	3		11		14	
I feel the future is hopeless			6		9	
**Group (Binary Ratings)**	**Total**	**Segment 1 of 2**	**Segment 2 of 2**			
**Base Size**	219	109	110			
**Additive Constant**	44	47	41			
**Strong for Mind-Set 1**						

I am in a very good financial condition	5	11				
Economically I struggle to afford life	5	10	1			
My financial situation is above average	3	9				
I have some economic problems	3	9				
**Strong for Mind-Set 2**						
I feel my future might be somehow negative	3		11			
**Not strong for either mind-set**						
The state health system covers all my medical expenses	1	1	1			
The state health system covers most of my dialysis treatment and most of other medical expenses	3	2	4			
The state health system covers most of my dialysis cost but only few other medical expenses		1				
No support is provided by the state health system	3	2	4			
My family strongly supports me	1		4			
My family supports me most of the time	1	3				
My family supports me only partially	2	2				

I do not have any support from my family	1		1			
I feel very encouraged about my future	1		7			
I feel my future might be somehow positive	1		7			
I feel the future is hopeless			6			
**Group (Binary Ratings)**	**Total**			**Segment 1 of 3**	**Segment 2 of 3**	**Segment 3 of 3**
**Base Size**	219			84	76	59
**Additive Constant**	44			48	46	37
**Strong for Mind-Set 1**						
I am in a very good financial condition	5			12	1	
Economically I struggle to afford life	5			11	2	1
My financial situation is above average	3			9		
I have some economic problems	3			9		
**Strong for Mind-Set 2**						
I feel my future might be somehow negative	3				14	
I feel very encouraged about my future	1				11	
I feel my future might be somehow positive	1				10	

I feel the future is hopeless					9	
**Strong for Mind-Set 3**						
The state health system covers most of my dialysis treatment and most of other medical expenses	3					16
The state health system covers all my medical expenses	1					11
The state health system covers most of my dialysis cost but only few other medical expenses						10
No support is provided by the state health system	3			1		10
**Not strong for either mind-set**						
My family strongly supports me	1				4	
My family supports me most of the time	1					1
My family supports me only partially	2				1	4
I do not have any support from my family	1				3	1
